# Embracing comorbidity: a way toward understanding the role of motivational and control processes in cannabis use disorders

**DOI:** 10.3389/fpsyg.2015.00677

**Published:** 2015-05-27

**Authors:** Janna Cousijn

**Affiliations:** Departments of Developmental and Experimental Psychology, Utrecht UniversityUtrecht, Netherlands

**Keywords:** cannabis use disorders, depression, anxiety, comorbidity, motivation, control

## Background

Although the general public perceives cannabis as one of the less harmful illicit drugs, the past decades saw a surge in treatment demands for CUDs (UNODC, 2014). Cannabis nowadays is the primary illicit drug of concern in drug treatment services across North America, Oceania and Africa (UNODC, 2014). The low perceived harms of cannabis use are reflected in the small number of studies investigating the neurocognitive processes underlying CUDs [e.g., only 3 published functional Magnetic Resonance Imaging (fMRI) studies in individuals with a diagnosed CUD compared to controls, contrasting more than 1000 studies in individuals with an Alcohol Use Disorder]. Most studies on the mechanisms underlying cannabis abuse, including my own, investigated heterogeneous groups of chronic or heavy cannabis users with various levels of cannabis use related problems, not groups with diagnosed CUDs.

Even though a substantial part of regular cannabis users will not experience any clear negative social and health consequences of cannabis, this does not imply that CUDs are less severe than other Substance Use Disorders (SUDs). The mental health issues associated with CUDs are substantial and often include comorbid psychiatric disorders including depression and anxiety (Stinson et al., 2006). Moreover, CUDs are difficult to treat and long-term abstinence is achieved by fewer than 20% (Danovitch and Gorelick, 2012). This urgently calls for a better understanding of CUDs. It is therefore time to reach out to those coping with CUDs by studying the mechanisms underneath. The goal of this opinion article is twofold: First, I want to address the strong need for neurocognitive studies in CUDs. Second, I propose that studying neurocognitive commonalities and differences between CUDs and comorbid disorders like depression and anxiety has great potential to unravel the mechanisms underlying CUDs and to eventually reveal new treatment targets.

## Motivational and control processes in cannabis use disorders

Strong motivations towards drug use (e.g., craving, automatic tendencies to attend to and approach the drug), paired with an insufficient capacity to keep these under control are thought to play a prominent role in SUDs (Goldstein and Volkow, 2002; Robinson and Berridge, 2003; Dawe and Loxton, 2004; Everitt and Robbins, 2005; Wiers et al., 2007; Verdejo-Garcia and Bechara, 2009). Recent behavioral studies suggest that this is also the case in CUDs: confrontation with cannabis or related objects and contexts (i.e., cues) can trigger craving (e.g., Gray et al., 2011; Lundahl and Johanson, 2011), capture their attention (attentional bias; e.g., Cousijn et al., 2013b; Asmaro et al., 2014), and activate approach tendencies (approach bias; e.g., Field et al., 2006; Cousijn et al., 2011). In addition, cognitive control-related functions like planning, organizing, problem solving, decision-making, and working-memory appear to be impaired in individuals with a CUD (Fernandez-Serrano et al., 2011). Chronic cannabis exposure may (temporarily) impair cognitive control, but cognitive control deficits may also be a risk factor for the onset of cannabis use and escalation into CUDs (Cousijn et al., 2014).

## Embracing comorbidity as a tool

**Figure 1:**
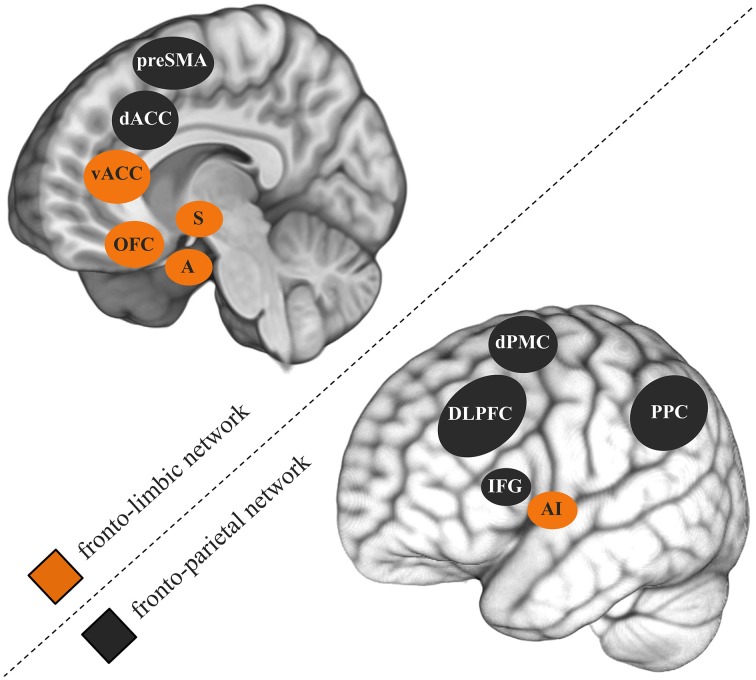
**Fronto-parietal and fronto-limbic brain networks thought to play an important role in cannabis use disorders, depression, and anxiety disorders**. preSMA, pre-supplementary motor cortex; dACC, dorsal anterior cingulate gyrus; vACC, ventral anterior cingulate gyrus; OFC, orbitofrontal cortex; S, striatum; A, amygdala; dPMC, dorsal premotor cortex; DLPFC, dorsolateral prefrontal cortex; IFG, inferior frontal gyrus; AI, anterior insula; PPC, posterior parietal cortex.

While the comorbidity between CUDs and other psychiatric disorders is widely accepted, neurocognitive studies mostly study disorders in isolation. Comorbid symptoms are often even controlled for by excluding such participants. A 3-year longitudinal epidemiological study specifically investigated the role of mental health factors in non-dependent versus dependent heavy cannabis use (Van Der Pol et al., 2013). Although externalizing psychiatric disorders like ADHD and conduct disorder were common to non-dependent and dependent users, internalizing psychiatric disorders such as mood and anxiety disorders were uniquely associated with dependence. Combined, the 283 almost daily cannabis users that participated in my previous studies revealed a correlation of *r* = 0.50 between cannabis use-related problems and depression symptoms (e.g., Cousijn et al., 2012; Beraha et al., 2013; Cousijn et al., 2013a,b). Similarly as in SUDs, neurocognitive models of depression (Weir et al., 2012) and anxiety disorders (Bruhl et al., 2014) stress the importance of dyscontrol over motivational processes and abnormal functioning of the underlying brain systems in the emergence of these disorders. Fronto-parietal and fronto-limbic brain networks are thought to play a key role in this (Figure 1; Seeley et al., 2007). The fronto-parietal network is thereby the main substrate for relatively *cold* executive control (e.g., working memory, attention, inhibition). The fronto-limbic network is primarily involved in emotion regulation, salience attribution and the integration of motivational information (e.g., reward, emotions) into decision processes.

The overlap in neurocognitive mechanisms underlying SUDs with depression and anxiety appears evident. Litle is known, however, about why certain symptoms cluster together and what differentiates disorders. From a clinical perspective, the vague boudaries between psychiatric disorders, the heterogeneity in psychiatric problems within patient groups and the poor treatment response in a substatial number of patients also underline the need to look beyond dichotomous disorder classifications (Casey et al., 2013). The new edition of the diagnostic statistical manual (DSM-V) introduced stages of disorder severity but still relies on self-reports (American Psychiatric Association, 2013). In the quest to identify more objective biomarkers for psychiatric problems, the US National Institute of Mental Health recently called for a transdiagnostic dimensional approach in the study of psychiatric disorders, in which the neurobiology underlying symptom dimensions is central, not the disorder classification itself (Casey et al., 2013). Embracing comorbid psychiatric problems, rather than factoring them out in neurocognitive studies, is an important step in this and I believe that such an approach has great potential to advance our knowledge of psychiatric disorders, including CUDs. An additional advantage of such an approach is that participants with comorbid problems are more representative of individuals with (sub-threshold) psychiatric problems in the general population and of patients in treatment.

Studying the common and unique neurocognitive mechanisms underlying psychiatric disorders may help us to identify new biomarker that could advance prevention and treatment. In the case of CUDs, we can only speculate about the neurocognitive mechanisms underlying CUDs, let alone understand why depression and anxiety disorders are associated with CUDs. Cognitive control deficits and malfunctioning of the underlying fronto-parietal brain networks may be shared between all three disorders, posing a general risk factor for the development of CUDs, depression and anxiety disorders (Koob and Volkow, 2010; Weir et al., 2012; Cousijn et al., 2013b; Bruhl et al., 2014; Peterson et al., 2014). In contrast, motivational processes within specific emotional and rewarding contexts may differentiate disorders. Although abnormal approach-avoidance behavior is common to all three disorders, depression and anxiety are associated with overactive avoidance of certain social and emotional situations (Trew, 2011; Caouette and Guyer, 2014), whereas CUDs may be associated with overactive approach of cannabis cues (Cousijn et al., 2011). Moreover, SUDs including CUDs and depression are both characterized by low positive affect (anhedonia) and abnormal reward responsiveness within various fronto-limbic brain areas (Koob and Volkow, 2010; Hatzigiakoumis et al., 2011; Elman et al., 2013; Morgan et al., 2013; Telzer et al., 2014). Interestingly, a recent PET study among 14 heavy cannabis users showed a link between anhedonia and reduced dopamine transmission in the striatum (Bloomfield et al., 2014). Unlike, CUDs, depression and anxiety further show abnormal processing of social emotional stimuli in the amygdala (Burghy et al., 2012; Weir et al., 2012; Caouette and Guyer, 2014). Further, amygdala connectivity with the ventral medial prefrontal cortex may differentiate between anxiety and depression by uniquely contributing to certain symptoms (Mcclure et al., 2007; Beesdo et al., 2009; Burghy et al., 2012).

Genetics are also known to play an important role in the risk for CUDs, depression and anxiety disorders. Motivational and control processes are influenced by genetic factors, including genes involved in drug metabolism and neurotransmission (Sweitzer et al., 2012). Motivational and control processes may thereby, at least partly, mediate the genetic vulnerability to all three disorders. For example the D2 dopamine receptor gene (DRD2) Taq1 A polymorphism affects dopamine binding in the striatum and is consistently associated with SUDs, depression and anxiety disorders (gene-disorder association indices retrieved from Gene Prospector; Yu et al., 2008). The A1 allele of the DRD2 Taq1 A polymorphism has been linked to reduced dopamine D2 receptor availability in the striatum, which could in turn reduce general reward responsiveness (Belcher et al., 2014). Another polymorphism consistently associated with all three disorders is COMTval158met (Yu et al., 2008). The COMT gene encodes an enzyme that is involved in the inactivation of catecholamine neurotransmitters like dopamine, epinephrine, and norepinephrine. The COMTval158met polymorphism has been linked to altered dopamine signaling in the prefrontal cortex, thereby influencing cognitive control (Bruder et al., 2005). Important to note CUDs, depression and anxiety disorders are polygenetic. Single genes are often only weakly associated with the risk for certain psychiatric disorders. To investigate genetic factors underlying polygenetic disorders large-scale multicenter genome-wide studies are needed. To allow DNA data contribution of small studies to large-scale consortia, DNA data collection should be facilitated for new studies, even though the primary objectives do not necessary comprise genetics. Moreover, epigenetics should be considered, that is the processes involved in long-term changes in gene expression that are heritable to daughter cells. Interestingly, a recent study in mice showed that a single epigenetic mechanisms (histone methylation of fosb) can influenced gene expression in the nucleus accumbens and induce depression and addiction like behavior (Heller et al., 2014).

### A critical note

Although knowledge of the common and unique neurobiology underlying comorbid disorders could identify biomarkers, researchers and clinicians should carefully evaluate and compare the clinical value of such measures for the individual patient. Our group-based findings may not necessarily translate to the individual. Also, neuroimaging techniques are expensive compared to questionnaires and neuropsychological tests. It is therefore important to explicitly test if certain neural indices explain unique variance on top of simpler (and cheaper) methods.

## Conclusions

The worldwide high treatment demands for CUDs, but the significant lack of studies investigating it warrant new studies that investigate neurocognitive functions in cannabis users with a clinically diagnosed CUDs. Uncovering the common and unique neurocognitive mechanisms and associated (epi)genetics underlying CUDs and highly comorbid disorders like depression and anxiety can provide valuable knowledge for improving current state-of-the-art treatments and for developing new neuroscience based interventions, such as neurocognitive training (e.g., approach-action retraining; Wiers et al., 2011), neuromodulation (e.g., stimulating brain areas involved in control; Ressler and Mayberg, 2007; Berlim et al., 2013; Da Silva et al., 2013) and pharmacotherapy (e.g., medication that enhances emotion regulation; Ressler and Mayberg, 2007; Sofuoglu, 2010; Mohler, 2012; Farb and Ratner, 2014). I reiterate that it is vital to study motivational processes and cognitive control in ecologically valid groups of individuals, that is, by including those coping with comorbid psychiatric problems.

### Conflict of interest statement

The author declares that the research was conducted in the absence of any commercial or financial relationships that could be construed as a potential conflict of interest.
